# Systematic review using a spiral approach with machine learning

**DOI:** 10.1186/s13643-023-02421-z

**Published:** 2024-01-17

**Authors:** Amirhossein Saeidmehr, Piers David Gareth Steel, Faramarz F. Samavati

**Affiliations:** 1 Computer Science Department, University of Calgary, 2500 University Dr., Calgary, Canada; 2 Haskayne School of Business, University of Calgary, 2500 University Dr., Calgary, Canada

**Keywords:** Systematic review, Machine learning, Active learning, Technology-assisted review

## Abstract

With the accelerating growth of the academic corpus, doubling every 9 years, machine learning is a promising avenue to make systematic review manageable. Though several notable advancements have already been made, the incorporation of machine learning is less than optimal, still relying on a sequential, staged process designed to accommodate a purely human approach, exemplified by PRISMA. Here, we test a spiral, alternating or oscillating approach, where full-text screening is done intermittently with title/abstract screening, which we examine in three datasets by simulation under 360 conditions comprised of different algorithmic classifiers, feature extractions, prioritization rules, data types, and information provided (e.g., title/abstract, full-text included). Overwhelmingly, the results favored a spiral processing approach with logistic regression, TF-IDF for vectorization, and maximum probability for prioritization. Results demonstrate up to a 90% improvement over traditional machine learning methodologies, especially for databases with fewer eligible articles. With these advancements, the screening component of most systematic reviews should remain functionally achievable for another one to two decades.

## Background

Published research is seeing exponential growth, with Bornmann and Mutz [1] finding a tripling in growth rates from 2 to 3% prior to WWII to approximately 8 to 9% in 2012. This results in a doubling of the scientific corpus for many fields every 9 years, a trend that reflects the steady increase in the number of researchers [2] and can be readily confirmed as having continued or even accelerated. Employing the date range filter of the online search platform Semantic Scholar shows that for topics as diverse as procrastination, entrepreneurship, and inflammatory bowel disease, indeed, more than half of the associated studies have been conducted in the last 9 years. Confronted with such academic fecundity, few systematic reviews can long espouse to be comprehensive with the concomitant challenge that updating them is becoming increasingly difficult. Academic search engines and databases remain poorly parsed or indexed, resulting in an exceedingly large number of false positives, often exceeding 30-to-1 [3, 4]. One solution is to simply narrow one’s focus by artificially limiting the scope, time range, or sources (e.g., journals considered). Such specialization can be valuable, but when it becomes a necessity to ensure that the systematic review or meta-analysis remains manageable, this is less than ideal. Choices are being dictated not by our needs, but by our time, resources, and technology.

Described as an “exaflood” of information, to cope with it researchers are exploring ways to enhance our ability to obtain and screen the literature. Among the most promising of these options is incorporating machine learning into systematic reviews, often called technology-assisted review (TAR). Machine learning can be trained to emulate researchers’ inclusion and exclusion decisions, described as human-in-the-loop (HITL) or, more specifically, researcher-in-the-loop (RITL) [5]. Only a fraction of the articles need to be screened before machine learning takes over, drastically reducing the time required. For example, van de Schoot et al. (2021) [5] review six machine learning options, though focusing primarily on ASReview. As they summarize, there are a wide variety of options in the process that need to be evaluated and refined on multiple datasets, from what information to include (e.g., abstract, title, author) to how it might be preprocessed to the classifying algorithms applied.

One of these areas of refinement is how to make the best use of the researcher, that is RITL. Presently, the Preferred Reporting Items for Systematic Reviews and Meta-Analyses (PRISMA) [6] is a frequently updated system that is incorporating machine learning. In deference to people’s limited time and cognitive capacities, PRISMA suggests a two-stage filtering process, what we describe as sequential or staged. As stage 1, the articles are screened, typically using title and abstract, and then only those that pass this stage have their full-text retrieved. As stage 2, based on the full-text, a second screening occurs where, as per PRISMA, “Reports Assessed for Eligibility.” This is an efficient compromise between quality and quantity, as often during a systematic review the number of initial articles identified can be immense, consistent with the exponential growth of many fields. Consistent with typical practice [7], the PRISMA guidelines emphasize machine learning during stage 1, abstract and title screening, as per the directive exclusive to this juncture: “If automation tools were used, indicate how many records were excluded by a human and how many were excluded by automation tools.” This results in machine learning being trained on best guesses based on the limited information available during stage 1, rather than the definitive decisions based on full-text that occur during stage 2.

As subsequently reviewed, we have several options about how else to integrate PRISMA with machine learning, including possible interactions among these alternatives. In order to determine the difference between these combinations and the optimal combination for completing the task, simulations based on three datasets from distinct domains are considered in this work. In summary, the aim of this paper is to explore 360 options for conducting systematic reviews that optimize the inclusion of machine learning, specifically (i) feature extraction, (ii) classifier models, (iii) query strategies, (iv) data types, (v) screening, and (vi) features. We find that screening done with spiral, alternating or oscillating processing, where screening frequently switches between stages, is superior to the traditional two-stage filtering process.

### Researcher-in-the-loop (RITL)

As mentioned, PRISMA advocates for a *sequential or staged* approach, resulting in machine learning trained on only stage 1 choices, rather than the superior stage 2 full-text screening. Though errors of inclusion and exclusion (i.e., commission and omission) can be reduced during stage 1, such as through in depth training of researchers and looking for consensus by employing several researchers, there is an inherent limit to how much titles and abstracts can inform. At no additional manual work, we have several options. The machine learning could be retrained on stage 2, allowing the identification of any rejected articles in stage 1 where the evaluation is substantially changed. Also, the screening could be done *spirally or oscillating*, where those that pass stage 1 are frequently re-evaluated in terms of full text, reducing errors of commission. This means that those classified as rejected would comprise those which were “Rejected in Title/Abstract Screening” as well as those that were later “Rejected in Full-Text Screening.” Those classified as accepted would be only those “Accepted in Full-Text Screening.”

### Query strategy

Associated with RITL and machine learning is what order to present articles to the researcher that accelerates algorithmic training. The fewer articles needed to reach stable solutions (i.e., where additional articles negligibly improve the algorithm), the more efficient the overall process. Sometimes described as re-prioritization [8], there is a variety of ordering options, from random selection to those with the greatest uncertainty to the least uncertainty to those with the newest information. Query strategies help balance the dataset, which is desirable as systematic reviews tend to be considered unbalanced in that there are a great many more rejections than acceptances [9]. We explore prioritizing articles most likely to be accepted (i.e., max probability), which will help balance the data, and those potentially with the most untapped information, which are most likely to speed learning (i.e., max uncertainty).

### Dataset expansion

Not every systematic review is equally amenable to machine learning [10]. Some may have clear inclusion and exclusion criteria with concomitant markers that readily differentiate, while other reviews are poorly specified and described. To assess the capability of different strategies, ideally they are assessed on multiple systematic reviews. Though there should be some variation in effectiveness among different datasets, we expect substantial robustness.

Furthermore, what defines a dataset can differ substantially. Presently, the PRISMA guidelines has the acquisition of articles sensibly occurring after title/abstract screening, being that they are often onerous to collect and are not used at this preliminary stage. However, increasingly the underlying articles can be quickly or automatically obtained, such as EndNote’s Find Full-Text feature or LibKey [11]. This provides a deeper information base for machine learning, potentially increasing its effectiveness. Other options are also possible, such as including associated keywords. We suspect that the more information provided, the better machine learning will work.

Consequently, the same systematic review dataset can be further subdivided. We can consider articles that have title/abstracts, which is the traditional baseline. We can also constrain the dataset to those articles with automatically obtained PDFs, establishing the benefit to machine learning of expanding its information base. Similarly, we can constrain the articles to all those with PDFs, which includes those manually obtained during full-text screening.

### Feature extraction

Feature extraction or vectorization deals with the preprocessing of the information to make it more amenable to machine learning. This is analogous to principal component analysis (PCA), where a large amount of data is reduced to more informative and less redundant dimensions. There are several options, of which we focus on two: Bag of Words (BoW) [12] and term frequency-inverse document frequency (TF-IDF) [13]. BoW creates a vector that reflects the frequency of words within a document. This is a straightforward counting, in this case the number of times each word occurs, making subsequent analysis much easier. Building on this, TF-IDF considers not only the frequency of the term within a document but also how many documents the word or term occurs in. If a term occurs in every document (e.g., such as indefinite articles), it limits its usefulness. TF-IDF weighting can help speed machine learning by emphasizing words that can potentially be informative. Given its greater sophistication, we expect TF-IDF to outperform BoW. In addition, rare terms that occur in only a few or even one document will have higher TF-IDF scores but have limited usefulness (i.e., applicable to only a few or one document). Consequently, we explore whether the range of document frequency can be constrained, where fewer terms can be processed.

### Algorithmic classification

Once the features of the data have been extracted, exactly how these elements will be analyzed remains to be determined. In this case, the appropriate algorithms are machine learning classifiers, of which there are at least eight major options [14] along with numerous variations. Here, we focus on four from those eight: statistical learning (i.e., naïve Bayes [15]), support vector machine (SVM) [16], logistic regression [17], and random forest tree [18]. These approaches have been shown to work favorably for systematic review, balancing speed, data requirements, and accuracy [9]. Naïve Bayes has often been used for text classification and builds readily upon the previous feature extraction. Essentially, it determines the conditional probability of features to determine the posterior distributions of whether an article should be included or excluded. Like naïve Bayes, SVM [19] works quickly and dependably with a limited dataset, making it useful for systematic reviews. SVM can be likened to running a regression in reverse, where instead of finding a line that minimizes its distance to the data points (i.e., least squares), it seeks a line (a hyperplane) that maximizes the margin between the two groups’ data points. Also, we have logistic regression, where once researchers have classified a sufficient number of cases, we simply use regression to create a predictive equation. Often, logistic regression is combined with lasso regression to cope with the common case where the number of features exceeds the number of cases. Finally, random forests is based upon decision trees, splitting the datasets by features, in this case terms. It attempts to find a decision tree that differentiates between those accepted and those rejected. We expect that all techniques will perform comparably but that there will be substantial differences in processing time.

## Methods

There are four parts of the methodology to be described. First, we consider how to incorporate RITL most effectively. Second, we apply our methods to three different datasets and review how we process them. Third, we vary different components of the process and run simulations on each variation. As per Table 1, these components are as follows: feature extraction (i.e., aspects of the text), classifier models (i.e., algorithms), query strategies (i.e., what articles are prioritized), data type (i.e., title/abstract versus full-text), screening (i.e., whether RITL decisions are based on title/abstract or full-text), and features (i.e., the corpus that feature extraction is based upon). Fourth, we review how we evaluate the outcomes of this simulation, focusing on increased productivity and computational efficiency.

**Table 1:** Simulation conditions

Criteria	Choice	Total Number
Feature extractions	TF-IDF high	3
	TF-IDF low	
	BoW	
Classifier models	Logistic regression	4
	Naïve Bayes	
	Random forest	
	SVM	
Query strategies	Max uncertainty	2
	Max probability	
Data types	Everything	3
	EndNote-only	
	Full-text	
Screening	Full-text screening	2
	Title/abstract screening	
Features	Title + abstract	3
	Title + abstract + EndNote	
	Title + abstract + EndNote + PDF	
Total		360

### RITL process

Screening in the systematic review process is one of the most time-consuming parts of the process. For a typical systematic review, researchers need to determine the relevancy of an article or study often for several thousand papers or more. Machine learning can help reduce this workload. A subset of machine learning is active learning, where a learning algorithm interactively prompts a user to label data with the desired outputs (e.g., include or exclude). In active learning, the algorithm proactively selects what instances (e.g., articles) should be annotated or labeled next by the user from the pool of unlabeled data. The fundamental strategy behind active learning is that a machine learning algorithm could potentially reach a higher level of accuracy while using a smaller number of training labels if it were allowed to choose the data it wants to learn from. This makes active learning part of the human-in-the-loop (HITL) paradigm [20], where it has been proven to be very effective [21]. Systematic review screening, which inherently has a person annotate instances (i.e., label articles as relevant or not), is readily convertible to an active learning process, in this case RITL (research-in-the-loop). The process is iterative as the dataset is incompletely labeled and the learning algorithm is sporadically re-informed by the choices of the researcher. How best to integrate an active learning algorithm in the systematic review process, that is how to reduce workload along with simultaneously finding most or all of the relevant articles, is advancing but yet to be definitively determined.

**Fig. 1:**
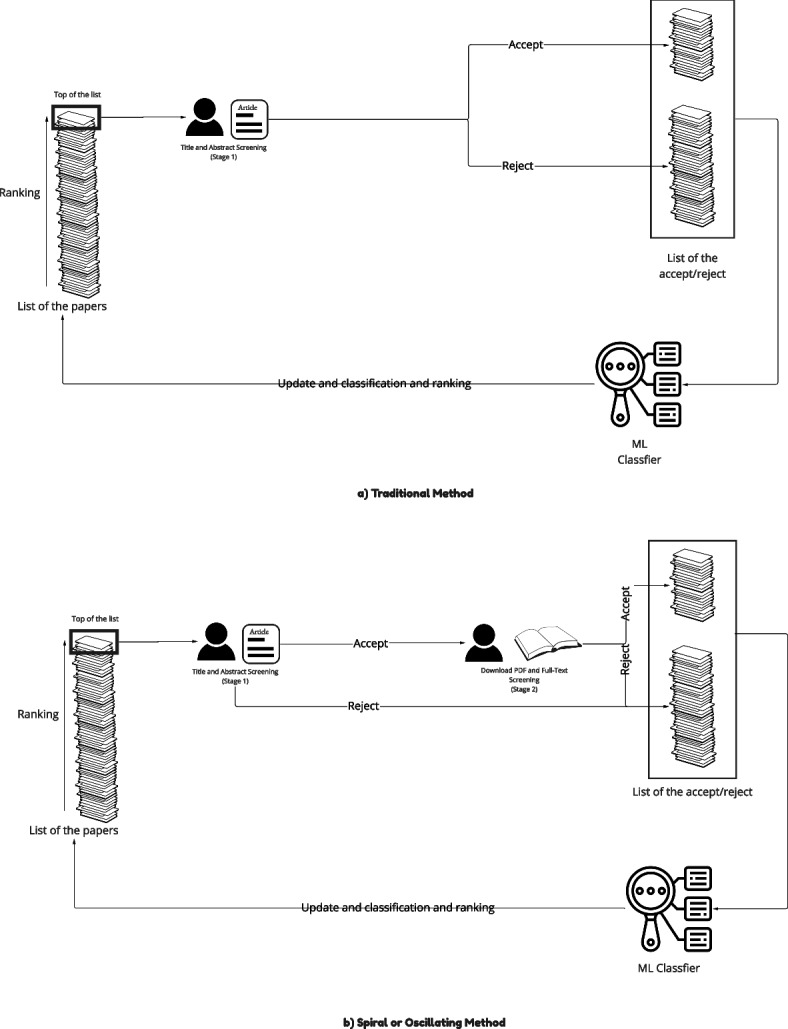
RITL process

This RITL process is reviewed in Fig. 1, of which we show a traditional method and a new spiral or oscillating method. Both of them represent RITL. Initially, the researcher randomly takes articles from the list of papers and then labels them “Accept” or “Reject.” When there is a sufficient number of articles labeled, the machine learning (ML) classifier attempts to predict whether unlabeled articles would be accepted or rejected by the researcher. Based on this, it also sorts the list of papers to prioritize those that would either be useful to the researcher (e.g., most relevant) or help speed its own learning. As can be seen, the process is circular, putting the researcher “in the loop.”

The difference between the traditional method and the spiral method determines what criterion active learning is trained upon. For the traditional method, it follows a classic two staged PRISMA process, where in stage 1 the researcher’s decisions are based solely upon the title and abstract. This reflects that it is excessively cumbersome for the researcher to examine each article’s full text, so only the subset that passes title/abstract screening is later evaluated, hence two stages. As mentioned in the introduction, the better criterion is based on full-text, which can provide a definitive decision based on full information (e.g., PDF of articles). To take advantage of this better criterion while acknowledging that the process needs to be manageable, we propose spiral screening. Rather than waiting until all the articles are title/abstract screened (which does not include the full-text screening information in the process) before moving to the second stage, spiral or oscillating screening rapidly shifts between the two stages. Consequently, aside from the order of operations, the researcher’s experience is identical for both staged and spiral. In the Fig. 1 spiral example, we have full-text screening nested between title/abstract screening and the ML classifier. Since only those articles that pass title/abstract screening are considered for full-text screening, it does not increase the workload but does provide active learning with a better criterion to train itself upon.

### Simulation details

Presently, it is unclear what is the best configuration of active learning components for systematic review. We could address this experimentally, by assigning individuals to each condition and assess their screening efficiency. However, even if we had just 30 people per condition, this would require approximately 10,000 participants, which is not obtainable. Instead, we take the standard approach and assess through simulation. The advantage is that we only need a few fully annotated datasets, each of which receives every condition stipulated.

**Fig. 2:**
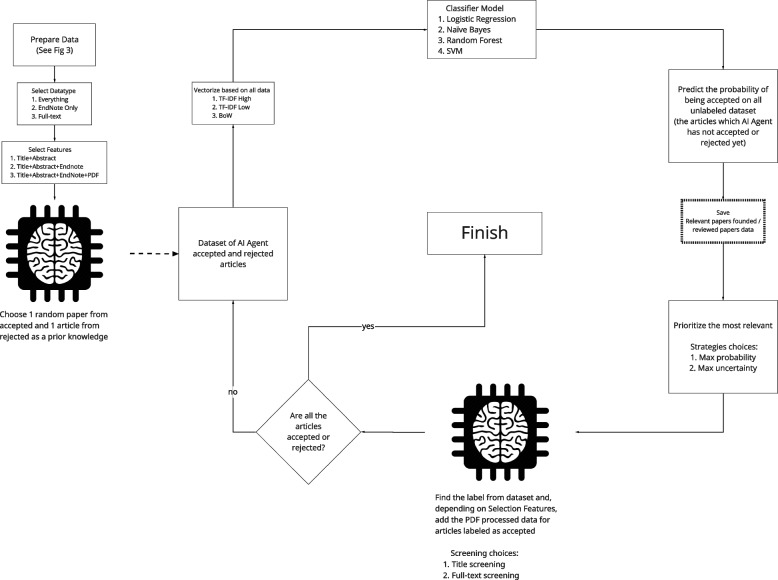
Simulation process

As shown in Fig. 2, the simulation begins with a prepared data set that can parsed into a variety of conditions. The simulation replicates the cyclical RITL process as per Fig. 1, but instead of an active researcher labeling the data, the simulation relies on an AI Agent that samples from previously established researcher decisions. Presently, we review the active learning algorithm conditions in addition to the information that could be included in the training process.

As per Fig. 2, the simulation process starts with selecting a dataset and then parsing it to align with one of our conditions. Having obtained access to the dataset, the AI agent selects an article randomly and labels it (which is simply drawing from previous researchers’ decisions). This acts as prior knowledge. Subsequently, the simulation vectorizes the chosen features using the selected feature extraction algorithm. This vectorized data is fed to the machine learning classifier, which trains itself based on it. The classifier algorithm then predicts the probability of acceptance for the unlabeled articles, which is saved. Based on the choice of the query strategy and the predicted probability for each unlabeled article, the simulation asks the agent to label a subsequent article, restarting the cycle, until there are no other articles left to be considered.

As per Table 1, we vary six different components of the entire process, resulting in 360 conditions per dataset, for a total of 1080 instances (i.e., 360 × 3 datasets). This is less than the 432 possible conditions as it is nonsensical to run the title/abstract screening condition with the full-text condition (i.e., those in the full-text condition have already been accepted during title/abstract screening). To address overfitting and assess stability, we run each instance ten times and take the average. Notably, this addresses both under and over performance of the algorithm due to processing less or more informative articles, though there will eventually be convergence as more articles are processed. As discussed in the introduction, we explore the following: feature extraction, classifier model, query strategy, data type, screening, and features. For feature extraction, as per the introduction, we consider BoW and TF-IDF. For TF-IDF, we limit it by document frequency (i.e., the total number of documents divided by the number of documents containing the term in the dataset). Necessarily, each term must have a document frequency ranging from 0 (i.e., it occurs in no documents) to 1.0 (i.e., it occurs within every document). Within this range, TF-IDF high is operationalized as having a document frequency between 0.1 and 0.9 and for TF-IDF low as between 0.2 and 0.7. If both perform equivalently, it suggests that we can further constrain TF-IDF’s range and achieve some reductions in computing resources required.

The algorithmic classification models (i.e., naïve Bayes, SVM, logistic regression, and random forest trees) were obtained from scikit-learn [22] python library, using their base parameters. For query strategy, maximum probability is rank ordering by the classifier model algorithm’s output. Maximum uncertainty is operationalized by scores midway in the range of the classifier model algorithm’s output. Data type reflects subsets of the three datasets, with transparent and self-explanatory descriptions. Everything is the intact dataset, with no restrictions. EndNote-Only are restricted to those with PDFs attached due to EndNote’s automatic find full-text option, and full texts are restricted to those which have been accepted during title/abstract screening and now have PDFs attached. Screening, as mentioned, is whether the article was accepted by the researcher based on title/abstract or full text. Finally, features are the columns in the dataset that were extracted, where we tested three nested levels of information. First, we limited ourselves to the traditional title and abstract. Second, we added those PDFs automatically obtained through EndNote. Third, we added the PDFs obtained during full-text screening.

**Fig. 3:**
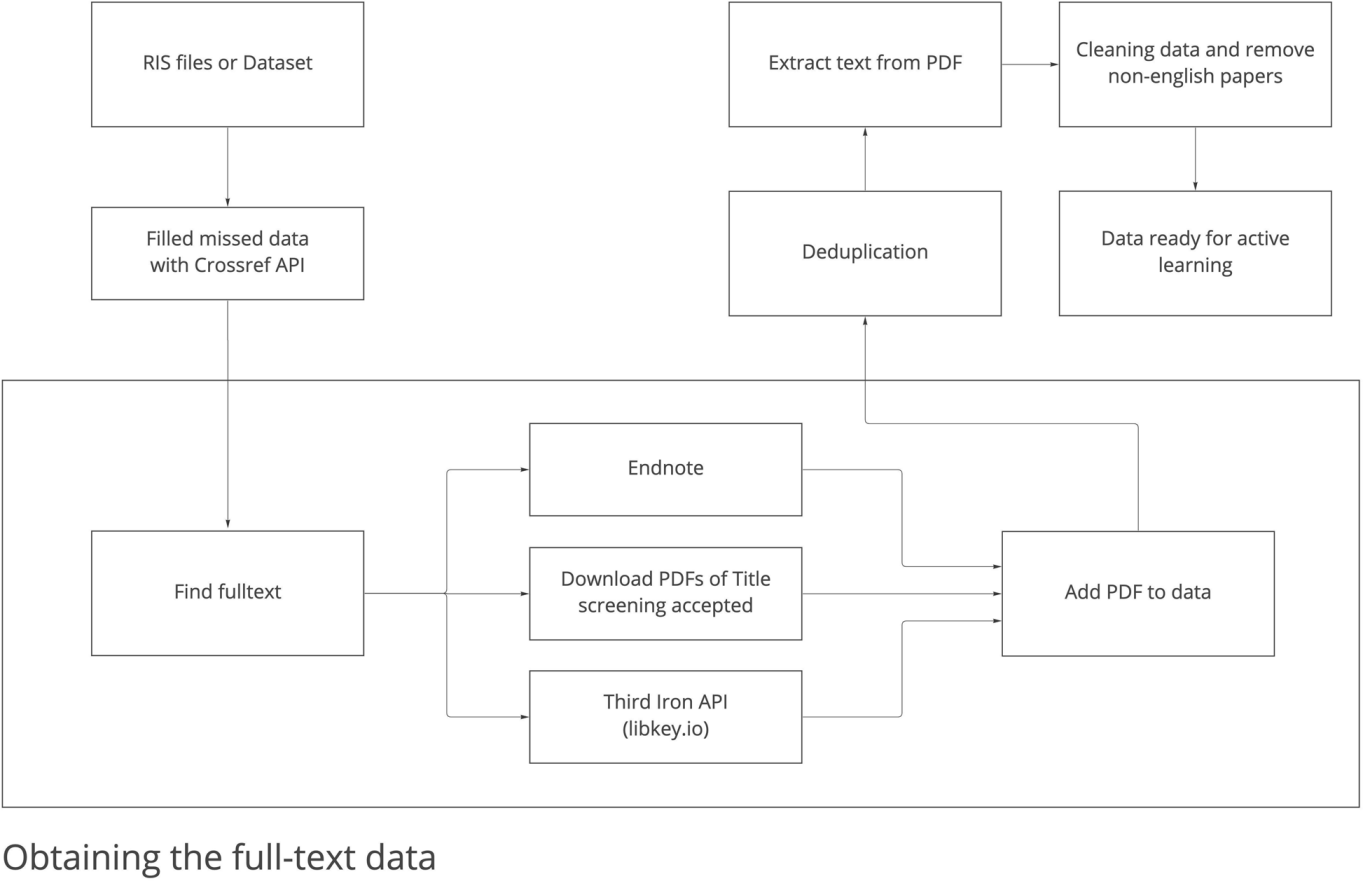
Dataset preparation

These six conditions were organized as per Fig. 2. The simulation emulates the review process, attempting to find the most useful operationalization of active learning in systematic review screening. Sensibly, we begin with dataset selection. After choosing and preparing one of the three datasets, as per Fig. 3, we select one of the 360 conditions. This begins with choosing a subset of the dataset or data type (e.g., only articles that have EndNote PDFs) and then features to be extracted (e.g., title + abstract + EndNote PDFs). Of note, it does not make sense to choose features extracted that are not contained within the “Data Type.” To enhance stability of our estimates, for multiple iterations, we use a random seed to determine the initial articles that the AI agent labels and then subsequently calculate the average of these iterations. If the features selected are title/abstract or title/abstract plus EndNote PDFs, these need to be vectorized only once. During PDF vectorization, to reinforce the importance of title/abstract, we include the previous vectorization of title/abstract to compensate for OCR limitations. However, if the features include full-text PDFs, which are added only after they are accepted in title/abstract screening, these must be periodically updated. The process ends after all the articles have been accepted or rejected. More information and details about the simulation can be found in our open source GitHub repository https://github.com/ammirsm/automatic-pancake.

### Datasets

The simulation is applied to three distinct datasets. The description of these databases is provided in Table 2. ASReview provides an open source github [23] where people archive their systematic review datasets, from which we obtained two. The cognitive behavioral therapy (CBT) [24] dataset reflects a meta-analysis of the outcomes for anxiety-related disorders and the post-traumatic stress disorder (PTSD) [25] dataset reflects a systematic review of PTSD studies that include latent trajectory analyses. In addition, we also had access to an ongoing systematic review on national culture, an update to a previous meta-analysis [26], which will be available in an Open Science Framework (OSF) folder upon publication. All datasets have both reviewers’ title/abstract and full-text screening decisions.

**Table 2:** Dataset description table

	Cultural project	PTSD project	CBT project
Total papers	12,107	6184	10,953
Total English papers	10,887	6152	10,933
Number of duplicates	2768	162	406
Full-text screening accepted	1379	43	73
Title abstract screening accepted	3080	388	806
LibKey founded	3355	1318	6719
LibKey open access	434	254	1530
LibKey founded PDFs	2916	1156	5902
Crossref founded metadata	3385	1375	7030
Endnote founded PDFs	2165	804	1388
Manually added PDFs	6055	327	698

These systematic review datasets have different properties, appropriately so as to determine the robustness of the process. This is often a byproduct of the search strategies and topic (which can make it easier or harder to locate desired articles) as well as tolerance for errors of inclusion. As per Table 2, the CBT and PTSD datasets have a full-text screening acceptance rate of approximately 0.7%, while the cultural project has an 11% acceptance.

The processing of these datasets is summarized in Fig. 3. After obtaining the initial dataset, such as the RIS file, available missing data was obtained through a CrossRef API (e.g., precise title). We then attempt to obtain PDFs, using automated and semi-automated methods. This is notable, as we initiate this step earlier on in the process than recommended by PRISMA guidelines, though in a realistic approach that is not labor intensive. First, we use our EndNote full-text finding tool, whose efficacy is dependent upon journal access which varies among universities. Then, we use the Third-Iron API (libkey.io) to determine how many of the articles are published in open scientific databases and how many of them can be located using libkey APIs. Due to the restriction of the Third-Iron API, automated PDF downloads from their website is not yet possible, but this can soon change. The results of these attempts are shown in Table 2. In addition to these two automated download methods, we manually downloaded all PDFs for articles that passed the title/abstract screening to see how successful machine learning might be if full-text screening is done spirally rather than staged. Notably, the original systematic review does not have to be done spirally for us to run our simulation.

To further maximize the chance of obtaining PDFs, we conducted deduplication after this step. Minor differences in the DOI (Digital Object Identifier) and title references can hinder EndNote’s automatic full-text finding. There were few duplicates in the CBT dataset and the PTSD dataset. However, there were 2768 duplicate articles in the dataset for the Cultural project, whose data has not been preprocessed. As per Table 2, this reduced the number of articles by 3 to 23%. This number was further reduced as our machine learning approach (e.g., BoW) is language specific, and consequently, we needed to limit the dataset to English-only (being by far the most common). To accomplish this filtering, we used the langdetect python module (Version 1.0.8), which is a python port of language detection library [27] with a 99% accuracy rate, filtering out the non-English ones.

We prepared both title and abstract as well as full-text PDFs for feature extraction. We utilized the pdftotext [28] package in Python (Version 2.2.2), an open-source alternative, to convert our PDFs to text, with an emphasis on text data. Then, we removed extraneous data (e.g., emails, URLs, designations or ranks, numbers) from our text to improve feature extraction (i.e., which focuses on terms). Continuing this, we removed all punctuation and recommended stopwords (i.e., words already identified as having limited usefulness for NLP) and tokenized the data, preparing it for feature extraction. As per Fig. 3, the data is now ready for active learning. The source code for each of these procedures is accessible in our GitHub repository for more information and explanation.

### Evaluation

There are various ways to evaluate the performance of the process, including the amount of data needed to operate and the time it takes to analyze that data. For systematic reviews, we focus on five key metrics. First, there is Work Saved over Sampling (WSS), which is the reduction in the number of articles needing to be screened [29]. Unless we have a perfect prediction, there will be a certain percentage of articles missed, so typically this is WSS for a given level of errors of omission, such as 5%. In other words, it is the number of articles that must be screened before 95% of the relevant total are found. Second, we are interested in relevant references found (RRF) [29], that is how accurate a machine learning option becomes after a given percentage of screened articles. For example, the number of relevant articles a machine learning technique can identify after screening just 10% of publications. Typically, RRF is demonstrated graphically, with relevant references found along the *Y* axis and the number of articles screened along the *X* axis. Third, we included precision, which is the number of true positives identified (i.e., the articles pre-identified as passing full-text screening, as per Table 3) divided by all the articles at WSS95%. Fourth, we consider accuracy, which is number of true positives + true negatives divided by total number of articles. For WSS95%, the true positives is fixed at 95% of all full-text screened articles accepted, as per Table 3. True negatives is the remaining articles at WSS95% minus 5% of all full-text screened articles accepted (again, as per Table 3). Fifth, we measure the retraining time required in seconds to reflect computational intensity as some machine learning techniques are far more computationally intensive and may only provide marginally better results.

**Table 3:** Differences among classifier algorithms for staged and spiral processing

	Staged			Spiral		
	Cultural	PTSD	CBT	Cultural	PTSD	CBT
Random forest						
WSS95%	6852	4552	4752	5502	702	7752
% of articles	76.50%	74.00%	43.60%	61.50%	11%	71.10%
Precision	19.12%	0.90%	1.46%	23.81%	5.82%	0.89%
Accuracy	48.49%	26.64%	57.14%	60.89%	89.22%	29.7%
Seconds required	116.6	62.4	78.5	1763	7.87	202.2
Logistic regression						
WSS95%	6802	4402	4952	5802	452	4252
% of articles	76%	71.60%	45.40%	64.80%	7%	39.00%
Precision	19.26%	0.93%	1.40%	22.58%	9.04%	1.63%
Accuracy	48.95%	29.08%	55.31%	58.14%	93.28%	61.71%
Seconds required	184.1	83.5	124	1841	23.4	155.9
SVM						
WSS95%	7052	4752	5502	6102	952	6602
% of articles	79%	77.20%	50.50%	68.20%	15.50%	60.60%
Precision	18.58%	0.86%	1.26%	21.47%	4.29%	1.05%
Accuracy	46.66%	23.39%	50.28%	55.38%	85.16%	40.22%
Seconds required	513.3	140.7	289	5750	11.78	300.5
Naïve Bayes						
WSS95%	8102	4502	5652	7052	1952	7202
% of articles	91%	73.20%	51.80%	78.80%	31.70%	66.10%
Precision	16.17%	0.91%	1.23%	18.58%	2.09%	0.96%
Accuracy	37.01%	27.45%	48.91%	46.66%	68.90%	34.73%
Seconds required	73.23	39.87	82.4	1668	37.55	187.5

For all conditions, query strategy = maximum probability and data type = everything. Staged processing represents screening = title/abstract screening and features = title + abstract. Spiral processing represents screening = full-text screening and features = title + abstract + EndNote PDF + [full-text PDF]

## Results

We implemented a system that simulates all 360 options and summarize it with a Tableau dashboard for interactive examination at: https://hubmeta.com/exploring-ai/. Each of the three datasets is displayed by default. To choose a specific condition, select the algorithm(s) from classifier (e.g., random forest), the query strategy from strategy (e.g., maximum probability), the text vectorization from feature extractor (e.g., BoW), a subset of the dataset from data type (e.g., EndNote only is all the articles that EndNote automatically located PDFs; title accepted are all those considered for full-text screening), type of screening decision from Screening (i.e., title/abstract screening versus full-text screening), and what data was vectorized from features (e.g., title + abstract + EndNote PDF + [full-text PDF]). The results can be shown as the raw actual values or the percentages, and, in either case, we have superimposed a line to indicate WSS 95% and RRF 10%. Again, not all combinations are sensical. For example, choosing title accepted for data type does not match with title/abstract screening (i.e., as only the articles previously accepted during title/abstract screening are being chosen). Also, if both title and full-text screening are selected simultaneously, they have different peaks, that is the total that would be approved during each stage, but we can display only one WSS 95% and RRF 10% cutoff. From this list of 360 conditions, we focus on those that address our core questions. We give the required settings for the Tableau dashboard to recreate our results, which is recommended for visualization as it provides clear identification regardless of the number of conditions contrasted.

**Fig. 4:**
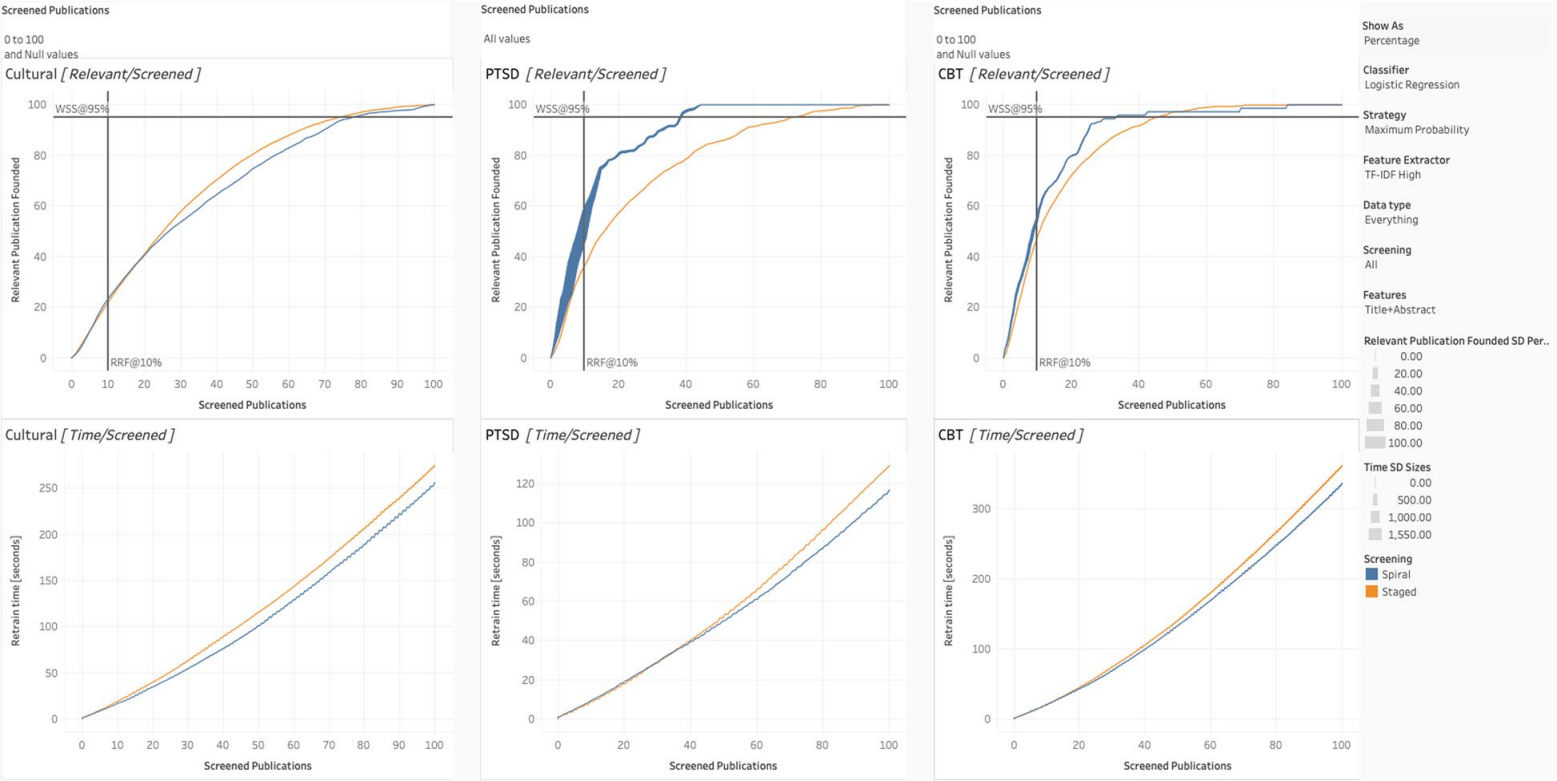
Staged vs. spiral processing

**Fig. 5:**
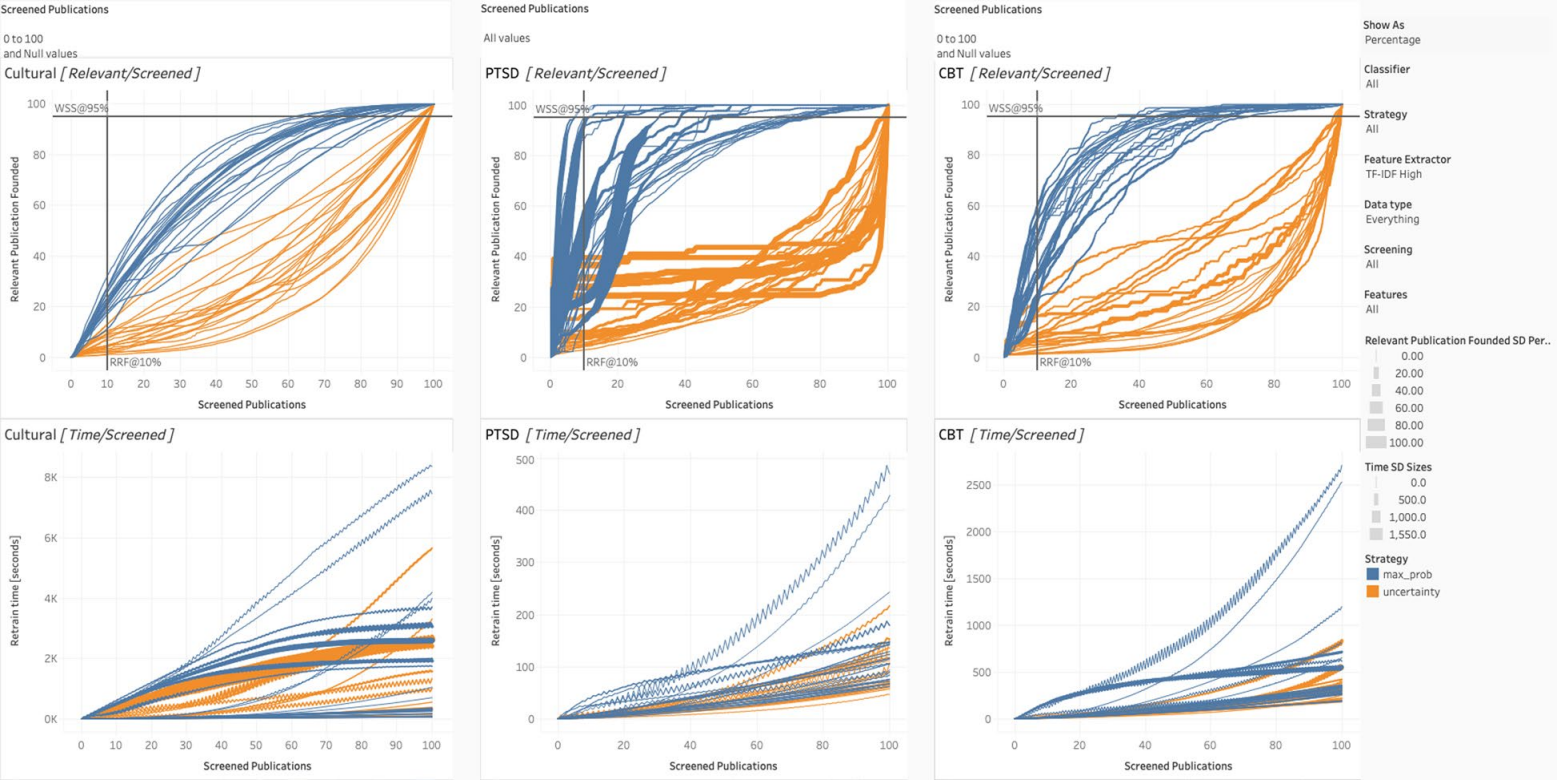
Maximum probability vs. maximum uncertainty

**Fig. 6:**
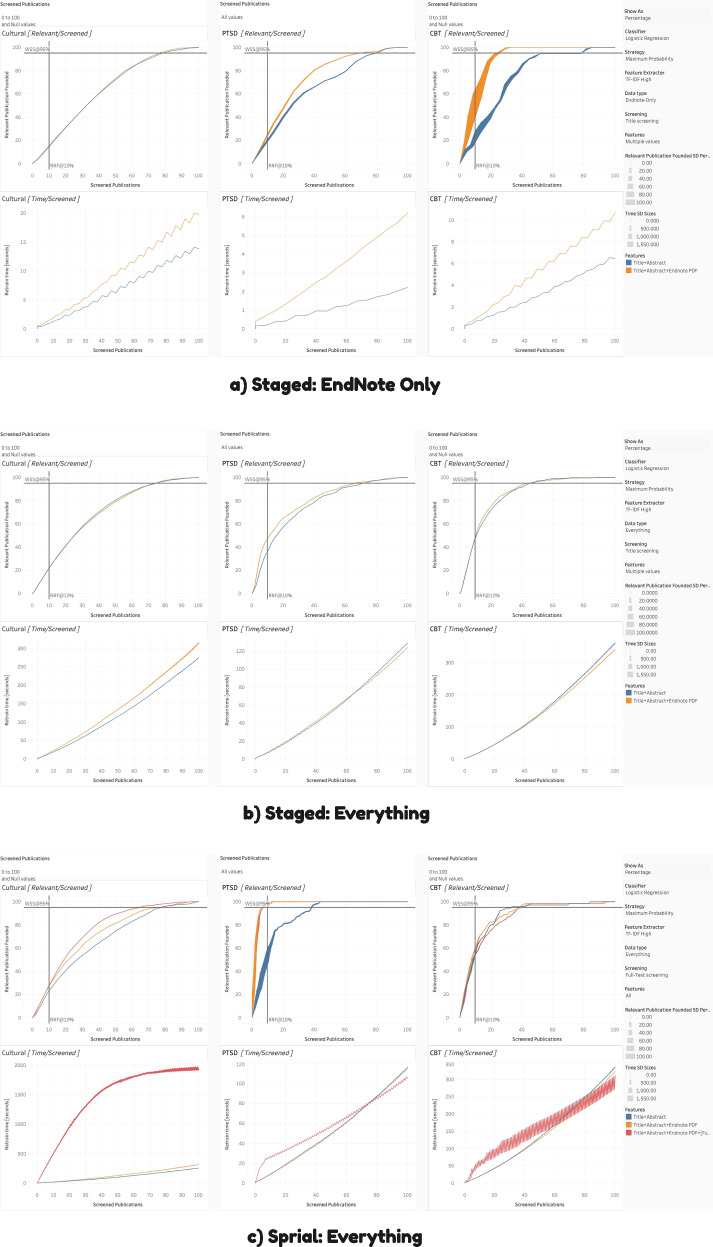
Dataset expansion

**Fig. 7:**
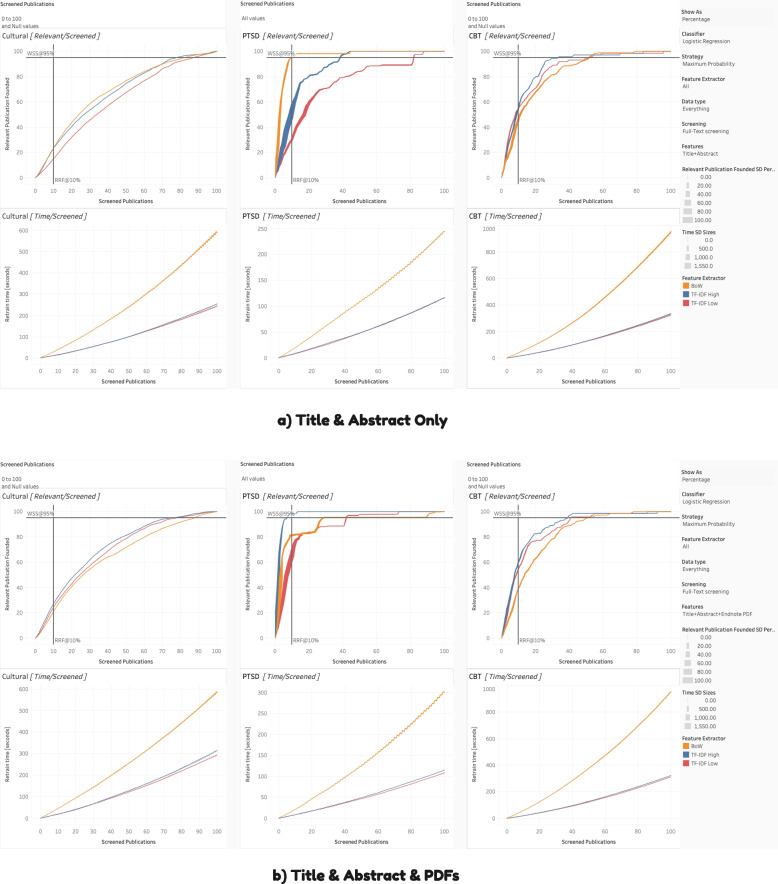
Feature extraction for **a** vs. **b**

**Fig. 8:**
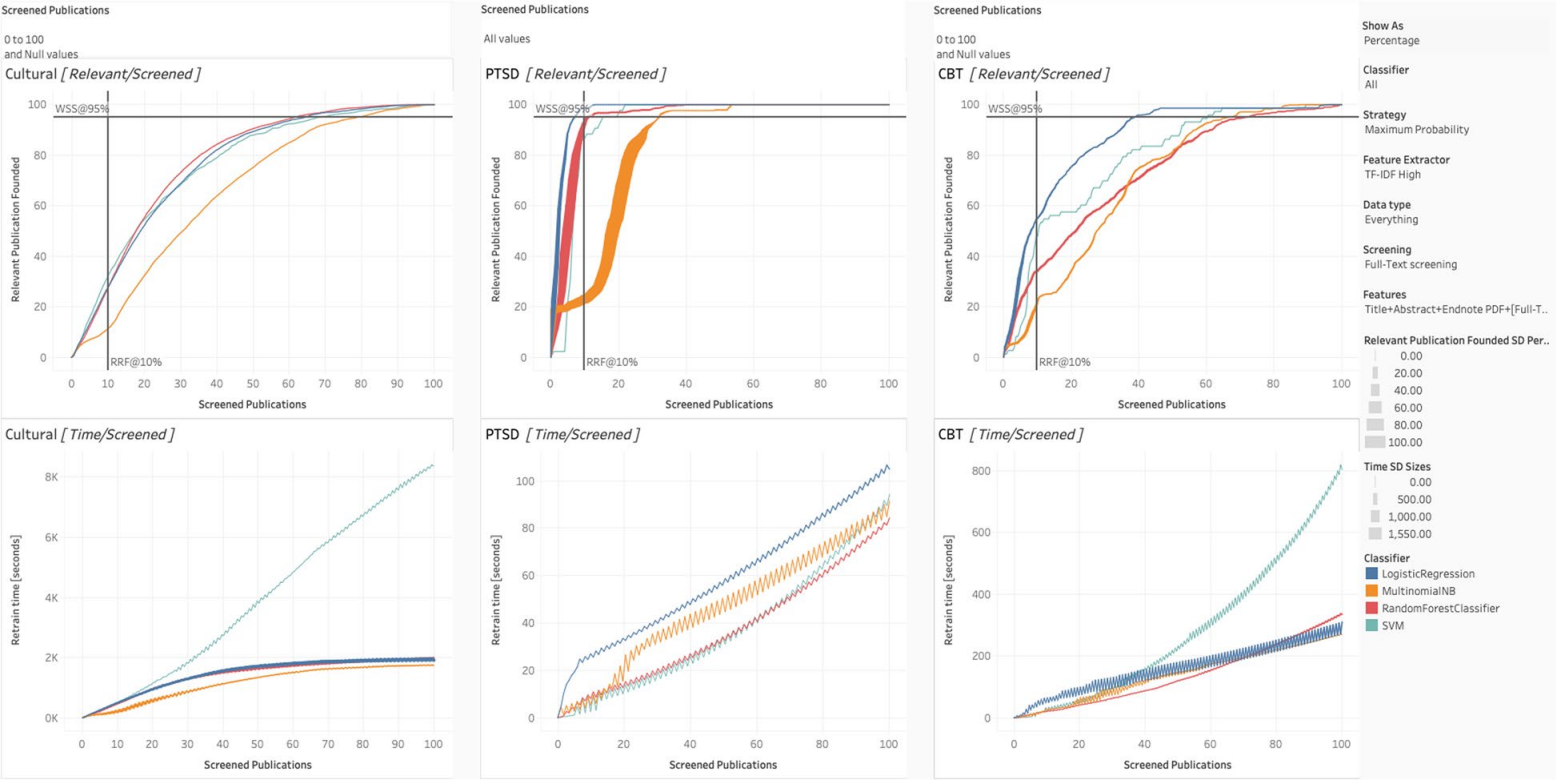
Classification algorithm for spiral processing

To interpret the Tableau charts (as per Figs. 4, 5, 6, 7, and 8), we review the following elements. The standard deviation reflects the percentage of the relevant articles found (e.g., 20 relevant articles out of a possible 50 would be 40%), based on the ten iterations for each condition’s simulation. Thicker lines indicate larger standard deviations. When the lines are relatively narrow, the results can be considered stable. Each of the three datasets has two associated charts, generating six in total. The top chart represents WSS, with the number [or percentage] of relevant publications on the *Y*-axis and the number [or percentage] of articles screened on the *X*-axis. Together, they show how many articles must be screened to obtain a given percentage or number of relevant articles and curves reaching the top of the chart quicker are better performing. Again, we include two lines to assist interpretation: the horizontal line is WSS95%, and the vertical line is RRF10%.

The bottom chart reflects the computational resources or processing time required. Like the top chart, the *X*-axis is the number of screened publications, but the *Y*-axis, in this case, is the time for retraining (in seconds). Those with steeper curves take more time and resources. Of note, at higher resolutions (i.e., shorter publication spans), these curves can display a jagged or zigzagged quality, which represents periodic re-vectorization, most prominent where new PDFs have their features extracted. To emphasize that this re-vectorization is intermittent, we report the term within square brackets (i.e., [full-text PDF]).

### RITL: staged versus spiral

To determine the difference between screening sequentially and spirally, there are two issues. When done spirally, not only is the criterion improved but there is more data to train the system (i.e., the accumulation of full-text articles). We focus first on the improved criterion and in a subsequent section on the “Dataset expansion” section.

To emulate the traditional staged process, we need to add two conditions together. First, reviewers conduct their title/abstract screening to WSS95%. Second, they then conduct full-text screening, to WSS95% or WSS100%, usually depending on the number of articles. Adding these two numbers together gives you the traditional staged process. To get stage 1 (title/abstract screening) in Tableau dashboard, set the classifier, strategy, and feature extractor to your preference. Set data type to “Everything,” screening to “Title [Abstract] Screening,” and features to “Title + abstract.” Note when the number of publications reaches 95%. To get stage 2 (full-text screening), use the exact same settings, except set data type to “Title Accepted” and Screening to “Full-Text Screening.” Add the numbers together. For example, for the PTSD dataset [25], it requires approximately 4500 to screen titles and 450 to screen full-text each at WSS95% (with variation depending on classifier and feature extractor), for a total of 4950 screenings.

To emulate the spiral process, replicate the same setting with these exceptions. Set the data type to “Everything” (which means every article is included, whether it has PDFs or not) and screening to “Full-Text Screening.” In this case, for the PTSD dataset [25], WSS95% can be achieved in 2350 articles, with no increase in the number of full-text screened (i.e., remains at approximately 450). Again, only those articles accepted on the basis of their titles and abstracts would be evaluated with their full-text. Without any additional manual work, we can reduce the title/abstract screening workload from 73% of the total (i.e., 4500) to 45% (i.e., 2,350 or an absolute reduction of 1750 articles) simply by processing them spirally. The reduction is due to both the improved criterion and because the articles most likely to have data are potentially being prioritized and presented first (i.e., setting the strategy to maximum probability). Running full-text screening spirally establishes when we have exhausted the supply of actually relevant articles, which happens more quickly as per Table 3. This is best exemplified by Fig. 4, which directly compares staged versus spiral processing.

### Query strategy

We considered two options for query strategy: maximum probability and uncertainty. Interpretation is straightforward. For all conditions, as per Fig. 5, maximum probability was superior, likely due to providing a more balanced training dataset. Consequently, we recommend that for the remaining contrasts, we set query strategy to maximum probability by default.

### Dataset expansion

Further following up on RITL, the typical PRISMA guidelines can be adjusted to increase the information available. To this end, we conducted several contrasts, as displayed in Fig. 6. First, what is the advantage of including the more easily obtainable EndNote PDFs during title/abstract screening? On the Tableau dashboard, set data type to “Everything,” screening to “Title [Abstract] Screening,” and features to both “Title + Abstract” as well as “Title + Abstract + EndNote PDFs”. As can be seen, benefits range from small to negligible. However, there is a limited number of articles with PDFs, as per Table 2. If we constrain our data type to “EndNote-Only,” it can more clearly demonstrate the effects of having every publication with full-text. In this case, the benefits can be substantial, especially for the CBT dataset. Each algorithm does substantially better when drawing on PDF data, especially the random forest and logistic regression classifiers.

Second, what is the advantage of obtaining all the PDFs for the articles that have been accepted in the title/abstract screening during spiral processing? This is using the maximum information realistically available, as it includes only papers that would be acquired during Full-Text screening anyway at approximately the time they are individually acquired. In this case, change the screening setting to “Full-Text Screening” and set features to “All.” This will allow the contrast between all three levels of data (i.e., from title + abstract to title + abstract + EndNote PDF + [full-text PDF]). Here, the differences range from negligible to massive, depending upon the dataset. Referencing Table 3, 95% of the relevant PTSD dataset [25] can be obtained after approximately 452 articles using spiral processing. Moreover, staged processing would take 4402 to achieve WSS95%.

### Feature extraction

As displayed in Fig. 7, we tested two major categories of feature extraction: BoW and TF-IDF. Furthermore, we considered two variants of TF-IDF, one high (where the document frequency range is 0.1 to 0.9) and low (where the range is 0.2 to 0.7). For BoW, its performance relative to TF-IDF was contingent on the features. When there was less information (i.e., Title + Abstract), it tended to perform well. However, when there was more information (i.e., the addition of PDFs, such as during spiral processing), it tended to perform worse than TF-IDF. In addition, BoW takes more processing time, given that it generates more words than TF-IDF. For TF-IDF high and low, as might be expected, they often perform similarly but TF-IDF High proves to be the better choice.

### Algorithmic classification

We test our algorithms under staged as well as spiral processing conditions, as shown in Fig. 8. For staged, we put the Tableau settings at “All” for classifier, “Everything” for data type, and “Title [Abstract] Screening” for screening. Features tend not to make a substantive difference in performance here. All the algorithms tend to perform similarly, except for naïve Bayes, which tends to perform slightly to noticeably worse (i.e., for the cultural dataset). However, naïve Bayes was also the most computationally efficient, requiring the fewest seconds to run. The most computationally demanding by far was SVM.

For the spiral processing condition, we changed the setting to “Full-Text Screening” in Screening and to “Title + Abstract + EndNote PDF + [Full-Text PDF]” in features. Consistent with staged processing, naïve Bayes was the most computationally efficient and SVM was the least. Compared to staged screening, there was larger differences among the algorithms. Of the algorithms, logistic regression performed the most dependably, though random forest did slightly better for the cultural dataset. Again, naïve Bayes was the worst performing.

## Discussion

As machine learning continues to evolve, so too will how best to combine it with systematic review. Traditionally, the process follows the staged PRISMA guidelines, where articles are first screened by title and abstract (stage 1), then by full-text (stage 2). Though this makes sense for a purely manual process and its inherent human constraints, it limits the advantages of machine learning. First, the criterion is based on the inferior title/abstract screening rather than the superior full-text screening. Second, it limits the information available for machine learning by relegating PDFs to later in the process. By taking a spiral approach, where full-text screening is done periodically in batches, we avoid both of these limitations. As tested here, by taking a spiral approach and obtaining PDFs earlier (e.g., using EndNote’s automatic full-text finder), we can substantially increase the utility of adding machine learning to systematic review without any additional workload of significance.

Notably, lessons learned from training machine learning with title/abstract screening do not necessarily transfer to machine learning that also incorporates PDFs. Consistent with the variation among the databases explored, the type and depth of information made available itself make a difference. Our recommendation is that systematic review should take a spiral process, which includes PDFs whenever available. Also, we recommend that machine learning as a default should use logistic regression as the classifier algorithm, instead of the commonly recommended naïve Bayes or SVM. Feature extraction should favor TF-IDF over BoW and query strategy’s prioritization should be based on maximum probability. However, if a staged process is still being employed, where PDFs are not being considered, then the BoW often works slightly better and the choice of classifier algorithm is more flexible, though we still do not suggest naïve Bayes and researchers should keep in mind that SVM is the most computationally expensive by far. Likely, BoW does not work well in conjunction with PDFs as there are far more words to consider, making the size of the matrix unwieldy, and it is sensitive to document length, which can differ substantially.

Though we have a limited number of databases to explore (i.e., three), it is clear that they make a difference. It appears that spiral processing is most accurate when the database has a very limited number of target articles. The reason being is that likely the number of target articles influences the number of total articles that must be screened. For example, the PTSD project has 43 eligible articles, as per Table 1, and requires 452 articles in the logistic regression condition to identify 95% these, as per Table 3, creating a ratio of approximately 10:1. On the other hand, the cultural project has 1379 eligible articles, needing 5802 articles to identify 95% of them, a ratio closer to 5:1. Despite the cultural project being more precise and having a higher yield rate (i.e., 5:1 rather than 10:1), the PTSD project can be concluded far more quickly. Consequently, only about the top 10% of the articles that passed title/abstract screening in the PTSD project needed to be screened in full-text (i.e., 452 out of 4402) to capture the eligible articles. By processing spirally, we can engage stopping rules far more efficiently, at least in some cases. Like using a metal detector to find needles in a haystack, the fewer the needles and the bigger the haystack, the greater the advantage over manual methods. Fortunately, this appears to be the most typical case (i.e., low yield rates) [4].

As per Table 3, overall the best combination of techniques for these datasets appears to be spiral processing with these active learning elements: feature extraction − TF-IDF high, classifier model **−** logistic regression, query strategy − max probability, features − title + abstract + EndNote PDF + [full-text PDF]. Using this methodology, screening time can be reduced substantially. Compared with no active learning, the workload can be reduced to 7% (i.e., for the PTSD dataset). Compared with other active learning configurations, the workload can be reduced by a further 90% (e.g., 452 articles required versus 4752 articles using staged processing with SVM). If this number is sufficiently small and manageable, researchers may choose to return to the articles they rejected during title/abstract screening and confirm their choices with a full-text search, further reducing errors of omission.

### Future works

Consistent with the rapid incorporation of machine learning into the systematic review, there is still a multitude of steps to consider [30]. First, it is clear that different datasets have noticeably different properties, such as the ratio of relevant/irrelevant articles. This may be due to the field, the topic, or simply the search strategy. This range of databases can expand even further as language translations are included (e.g., from Mandarin to English). If robustness in results continues to endure, this becomes less of a concern. Otherwise, we could diagnose what machine learning conditions work best for a particular type of database. Instead, we still must rely on what works the best ceteris paribus.

Second, given that systematic review datasets are often unbalanced, it makes good sense to prioritize articles likely to be included. However, we could organize the process to speed learning in other ways, such as identifying those articles with the least information and prompt the gathering of the associated PDFs earlier, providing more information for machine learning. Related to this, we can expect the ease of article acquisition to continue to improve, as per the Open Access Movement [31]. At present, obtaining PDFs en masse is intermittently challenging, but this is not a technological issue as much as a business decision by publishers and this limitation could change rapidly. As an intermediate step, almost all the articles can be preprocessed by publishers and we will need to evaluate the degree of improvement this provides. If this continues to be sizeable, ideally all articles are preprocessed by the publishers or at least published in machine readable form, such as XML. Similarly, other information can be added to machine learning, such as exact authorship (e.g., not name but orcid.org nomenclature). A researcher who has previously published relevant articles is far more likely to publish again.

Third, as the databases increasingly have PDFs attached, different feature extractions may prove inconsistently useful. For example, there are dozens of BERTs (Bidirectional Encoder Representations from Transformers) [32], and they continue to be fine-tuned. When and to what degree these refinements provide improvement will need to be established. In addition, instead of using just NLP technology, we could employ image processing, useful for detecting graphs, tables and matrices [33].

Fourth, stopping rules are still being developed [34]. Ideally, a researcher would be informed when they have likely passed WSS95%. To help develop more precise stopping rules, a fully processed dataset, where every article is full-text screened would be ideal. Such a fully evaluated dataset provides an additional option to test for RITL: immediately proceeding to full-text. Running screening spirally helps to prevent errors of commission, whereby approved articles are immediately checked for eligibility. However, errors of omission, articles that were not approved for full-text screening, are not further assessed and thereby potentially errant. Described as true recall [8], we would like to know exactly how many articles the process missed and what they were. By taking a well-selected sample of articles for immediate full-text screening, skipping title/abstract screening entirely, we have an even better criterion with which to train as well as assess machine learning. As soon as the machine learning algorithm stabilizes, the remaining articles are screened automatically, whereupon traditional full-text screening resumes. In fact, we would want to create an extended set of such fully evaluated databases, providing a baseline that any new machine learning conditions can be run with. In this way, we would be able to easily make comparisons across studies instead of just within them.

Finally, this entire process can be iterative and used to improve the original search [35]. Based on the terms and other features extracted, a report can be given to the researcher summarizing what best identified the target articles. These may be used to compose additional search strings. Ideally, this step would also be automated, where search strings are generated that prioritize including the target articles and then attempt to exclude those rejected. Any additional articles detected with the revised search strategy are also then screened.

## Conclusion

The incorporation of machine learning tools into systematic review is rapidly advancing, making it a challenge to incorporate definitively in guidelines. Despite or even due to this speed, as findings are replicated across multiple databases from different fields, we expect that the optimal set of machine learning parameters will be soon established or at least a more limited set of options to focus upon. Until then, researchers should remember that the PRISMA guidelines are exactly that, evolving recommendations that allow for flexibility. The advantage of using machine learning is science-wide, accelerating research across virtually all fields. Our research is one of many steps toward this end though we hope a significant one.

## Data Availability

The codebase of the current study is available in the GitHub repository, https://github.com/ammirsm/automatic-pancake.
